# An Unusual Case of Complicated Urinary Tract Infection: Emphysematous Cystitis

**DOI:** 10.7759/cureus.13590

**Published:** 2021-02-27

**Authors:** Wahab A Gbadamosi, Aisha Miller

**Affiliations:** 1 Medicine, Coliseum Medical Centers, Macon, USA

**Keywords:** emphysematous cystitis, hematuria, urinary tract infection, urinary system obstruction

## Abstract

Emphysematous cystitis (EC) is a rare disease of the urinary bladder, caused by gas-forming bacteria, which can become life-threatening without appropriate evaluation. This report describes the case of a 77-year-old male with uncontrolled diabetes mellitus type II, who presented with suprapubic pain associated with frequency, urgency, dysuria, and gross hematuria involving the passage of clots. A review of systems was negative for weight changes, history of malignancy, urolithiasis, exposure to industrial chemicals, history of gastrointestinal tract disease, radiation therapy, and trauma. The patient was febrile upon admission. Laboratory findings were significant for lactate (2.7 mg/dl), and leukocytosis (28,100/uL). Computed tomography of the abdomen and pelvis showed dense material and air within the bladder, bilateral hydronephrosis, and hydroureter. He was managed with ceftriaxone (2 gm every 24 hours for 14 days), and a urinary catheter. EC should be considered as a differential diagnosis in diabetes mellitus patients presenting with hematuria, because knowledge of this rare finding may lead to early diagnosis and appropriate management.

## Introduction

Gas-forming infections of the urinary tract are a potentially fulminant life-threatening condition that requires prompt evaluation and management [1]. Gas-forming infections of the urinary tract are sporadic and may involve any part of the urinary system [2]. Emphysematous cystitis (EC) is a rare and distinct complication of the lower urinary tract, characterized by a primary infection of the urinary bladder, along with air within the bladder wall and lumen [1]. EC was first discovered via autopsy in the late 1800s by Eisenlohr, and there has been a recent increase in reported cases due to the wider use of abdominal imaging and a greater awareness of this unusual disease [3, 4]. The central role of urinary tract infection (UTI) in the pathogenesis of EC was established during the 30 years after Eisenlohr’s initial discovery, however, the pathogenesis of EC is still poorly understood. It is known that elevated tissue glucose in diabetes mellitus does provide a microenvironment for gas-forming microbes [3, 5]. Clinicians should suspect EC in diabetes mellitus patients presenting with urinary tract infection symptoms, hematuria, and altered mental status, because knowledge of this rare finding may lead to early diagnosis and appropriate management.

## Case presentation

A 77-year-old male with uncontrolled diabetes mellitus type II presented to our hospital with sudden onset hematuria and altered mental status. He was in his usual state of health, until about a week before the hospital presentation when he began to have generalized body fatigue. Symptoms developed into supra-pubic discomfort associated with frequency, urgency, dysuria, and gross hematuria involving the passage of clots. A day before the hospital visit, he was alert, oriented, and able to converse normally, but on the day of admission, he had been delirious and agitated for few hours. A review of systems was negative for weight changes, history of malignancy, urolithiasis, exposure to industrial chemicals, history of gastrointestinal tract disease, radiation therapy, and trauma.

Past medical histories were significant for coronary artery disease, diabetes mellitus type II, hypertension, benign prostatic hyperplasia, and gastroesophageal reflux disease. He had had coronary artery bypass surgery, a total metatarsal amputation due to chronic recurrent osteomyelitis, and angioplasty with stent placement in the lower extremity. There was no known drug allergy and home medications were atorvastatin, clopidogrel, metoprolol succinate, glargine, liraglutide, losartan, tamsulosin, famotidine. He resided in a house, had a 35 pack-year cigarette smoking, drank alcohol occasionally, and denied recreational drug use. Family history was significant for diabetes mellitus II, ischemic stroke, and transient ischemic attack.

On examination, the patient’s temperature was 100.6°C, his heart rate was 90 beats per minute, blood pressure 143/94 mm Hg, respiratory rate 18 beats per minute, and oxygen saturation 99% on room air. He appeared obese, confused, and agitated. He was alert to self, but not to time, place, or situation. The patient’s pupils were round and reactive to light. He was anicteric and dry mucosal, with a supple neck. The patient’s lungs exhibited decreased bilateral breath sounds due to body habitus. His heart had a regular rate and rhythms, as well as normal S1 and S2 sounds, with no gallops, rubs, or murmur. The abdomen displayed normative bowel sounds, was soft, and non-distended. There was no guarding, but there was tenderness at the suprapubic and costovertebral regions. Anal-rectal sphincter tone was normal and there was no mass in the rectum. The patient’s prostate was diffusely enlarged with normal palpation and the genitourinary examination showed gross hematuria, with clots. Testes were bilaterally descended with minimal atrophy. The patient’s extremities showed no ankle pitting edema. The results of the remaining examinations conducted were unremarkable.

**Figure 1:**
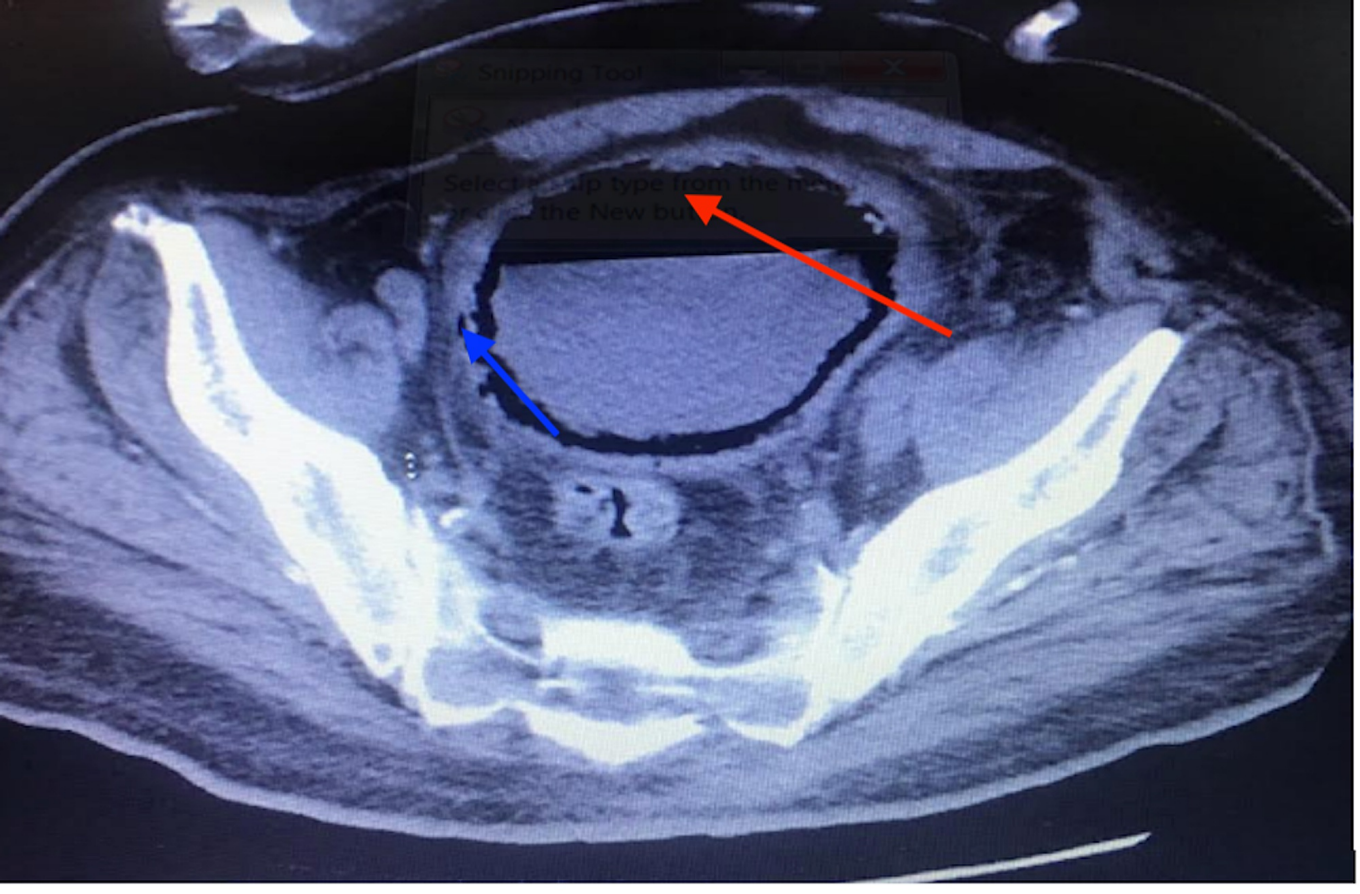
Axial CT demonstrating the presence of air in the bladder wall (blue arrow) and bladder lumen (red arrow)

**Figure 2:**
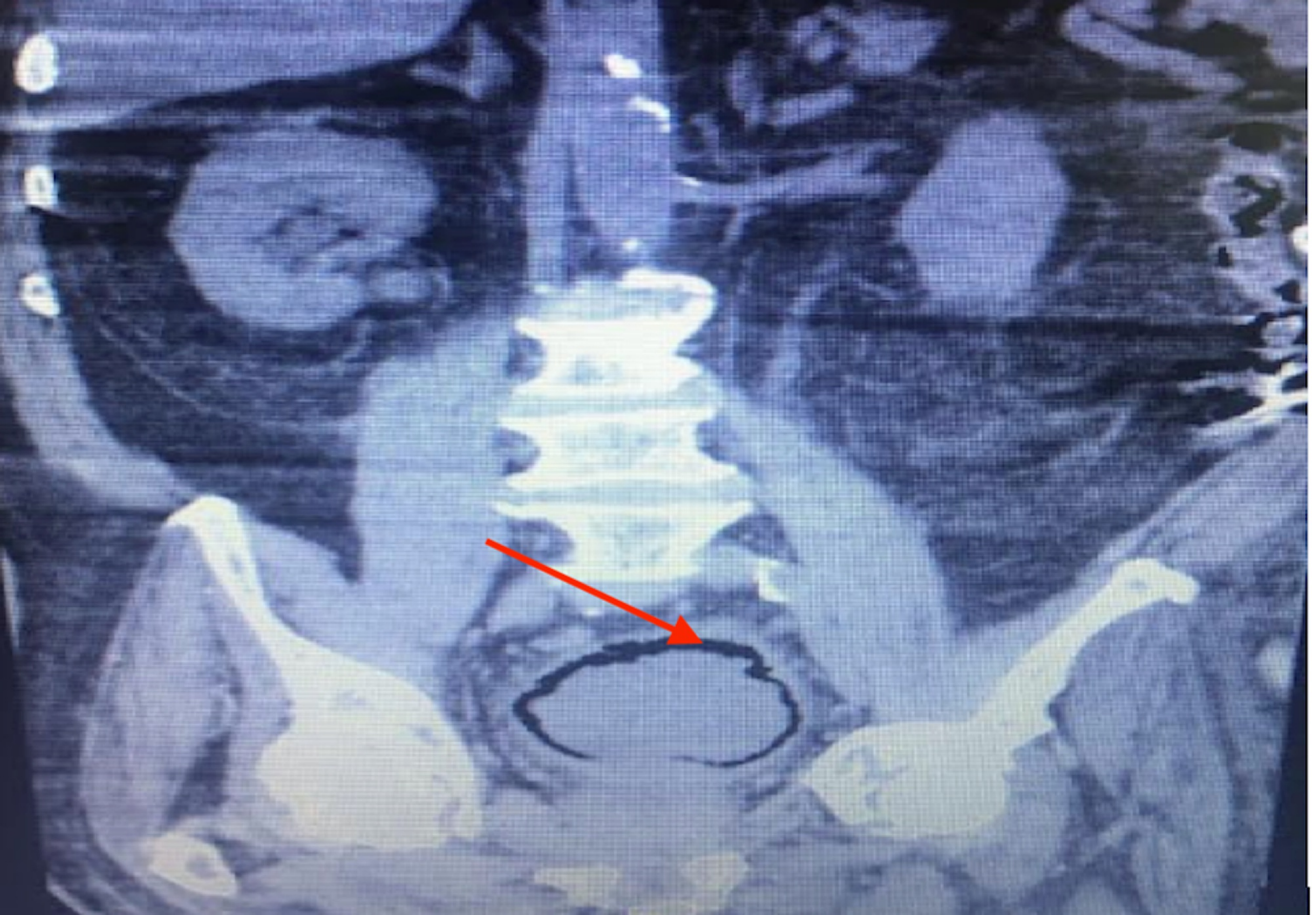
Coronal view demonstrating the presence of air in the bladder lumen (Red arrow)

**Figure 3:**
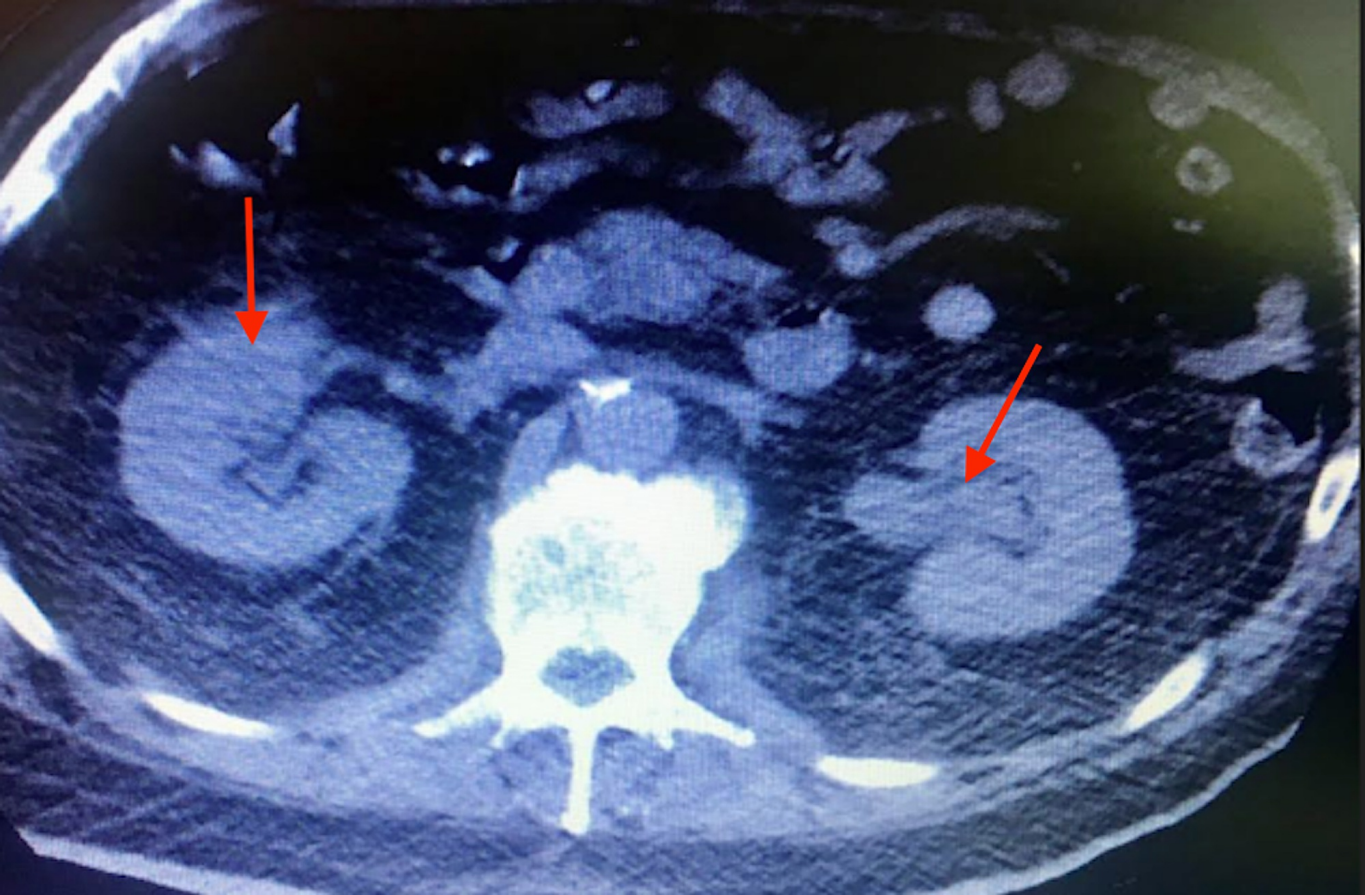
Axial view showing bilateral hydroureter and hydronephrosis (Red arrow)

Laboratory findings were significant for elevated lactate (2.7 mg/dl) (0.5-1.9), leukocytosis (28,100/uL), hemoglobin (Hgb) (11.1 gm/dl), hematocrit (HCT) (33.7%), and platelets (223 K/uL). The comprehensive metabolic panel was unremarkable, except for serum creatinine (1.6 mg/dl) (0.7-1.3), glucose (270 mg/dl) (74-106), and HbA1c (7.7%). Urinalysis revealed macroalbuminuria (>300 mg/dl), glucosuria (>1000 mg/dl), and ketonuria, leukocyte esterase, and leukocyturia (25-50 white blood cells per field, and too-numerous-to-count red blood cells per field). Prothrombin time (PT), partial thromboplastin time (PTT), international normalized ratio (INR), and ammonia levels were all normal. Electrocardiograph and chest X-ray results were both unremarkable. Computed tomography (CT) of the head was negative for acute pathology. CT of the abdomen and pelvis revealed dense material within the bladder, and air within the bladder wall, bilateral hydronephrosis, and hydroureter (Figures 1-3).

The patient was fitted with a urinary (Foley) catheter. Blood and urine cultures were obtained, and broad-spectrum antibiotics of intravenous piperacillin-tazobactam and vancomycin were started. Throughout the hospital course, specialists in infectious disease and urology were consulted. Concerns for pyelonephritis, nephrolithiasis, urolithiasis, malignancy, gastrointestinal tract, and central nervous etiology were all low after extensive investigation. The patient’s symptoms were caused by urinary outlet obstruction in the setting of enlarged benign prostatic hyperplasia. After two days, the blood culture was negative, but the urine culture grew Escherichia coli (10,000-50,000 CFU/ml), sensitive to ceftriaxone. The antibiotic was de-escalated to ceftriaxone. The patient’s mental and clinical status improved on day 6. He was discharged with outpatient intravenous ceftriaxone (2 gm every 24 hours for 14 days), a urinary (Foley) catheter, and outpatient follow-up. At his three-week visit, the patient’s urinalysis and culture were normal. Pelvic sonography revealed normal bladder wall thickening and no intraluminal gas.

## Discussion

Emphysematous cystitis (EC) is characterized by the presence of air localized in the bladder due to gas-forming bacteria. The prevalence and incidence of EC are unknown, but the overall death rate is 6.6% [6]. Thomas et al. reviewed 135 cases of EC, where the reported median patient age at presentation was 66 (0.11-90) years, and two-thirds of all cases had diabetes mellitus [1]. The risk factor for EC is primarily diabetes mellitus, however, other risk factors can include neurogenic bladder, urinary tract outlet obstruction, indwelling urethral catheters, and immunodeficiency [7]. Our patient belongs to the high-risk group for emphysematous cystitis, given his age, history of diabetes mellitus, and benign prostatic hyperplasia.

EC is usually due to Escherichia coli or Klebsiella pneumonia, but other causative organisms such as Proteus, Clostridium, Enterococcus, and Pseudomonas can cause EC [8]. In addition to the above infectious etiology, the effect of diabetes mellitus on the urinary tract includes diabetes nephropathy, renal papillary necrosis, and neurogenic bladder. These factors, when combined with glycosuria and impaired leukocytes, can lead to complicated urinary tract infections such as EC [1]. Our patient’s urinalysis showed macroalbuminuria, glucosuria, leukocyte esterase, leukocyturia, too-numerous-to-count red blood cells per field, and a urine culture that grew Escherichia coli (10,000-50,000 CFU/ml).

Emphysematous cystitis is often diagnosed with abdominal imaging. However, obtaining imaging in patients with UTI is not usually done, and this can result in an underdiagnosed number of EC cases [1]. A delay in diagnosis can cause bladder rupture, septicemia, and peritonitis. Failure to recognize this condition early in the course of the infection increases the associated mortality rate by up to 10% [6]. In our patient, a CT abdominal with contrast was used to guide the diagnosis.

Emphysematous cystitis, although rare, is potentially fatal if not treated properly [6]. The treatment of EC generally consists of antibiotics, bladder drainage, and glycemic control [1]. Endogenous antibiotics such as fluoroquinolones, beta-lactamases, and third-generation cephalosporins are some of the appropriate treatments [9]. Most patients in the 135-case report from Thomas et al. show that 90% of patients were treated with medical management, while 10% were treated with both medical and surgical intervention [1]. In addition to these therapy options, the treatment of diabetes is extremely important because it will reduce recurrence rates. Our patient was initially treated with a broad-spectrum antibiotic and placed on a urinary catheter. Once medically stable, he was discharged home with ceftriaxone 2 gm IV every 24 hours for 14 days.

## Conclusions

Emphysematous cystitis is a rare, gas-forming infection of the urinary bladder that is not often diagnosed by a routine approach. As per clinicians, a high degree of EC suspicion should be exercised when patients suffering from diabetes mellitus and urinary tract obstruction present with urinary symptoms and altered mental status. Due to the potential mortality associated with EC, early detection, imaging, and treatment are important for a favorable prognosis.
